# The Immature Infant Liver: Cytochrome P450 Enzymes and their Relevance to Vaccine Safety and SIDS Research

**DOI:** 10.7150/ijms.114402

**Published:** 2025-04-28

**Authors:** Gary S. Goldman, Richard Z. Cheng

**Affiliations:** 1 Independent Researcher, P.O. Box 444, Bogue Chitto, MS 39629; 2 Orthomolecular Medicine News Service, Cheng Integrative Health Center, 6149 St. Andrews Rd. Columbia, SC 29212

**Keywords:** CYP450 enzymes, vaccine safety, infant vaccination, sudden unexpected infant death (SUID), sudden infant death syndrome (SIDS), Neurological Developmental Disorders (NDDs), cytochrome P450, pharmacogenetic screening

## Abstract

**Aim and background:** Vaccines are a cornerstone of modern medicine, significantly reducing morbidity and mortality worldwide. Their administration in infants requires consideration of physiological maturity. Cytochrome P450 (CYP450) enzymes, crucial for drug metabolism, are underdeveloped at birth and mature over the first two to three years of life. While vaccines are not directly metabolized by CYP450 enzymes, emerging evidence suggests that certain excipients—such as polysorbate 80 and gelatin—could interact with CYP450 pathways, particularly in genetically susceptible infants. This study integrates pharmacogenetics and epidemiology to examine how CYP450 immaturity and variability may influence vaccine excipient metabolism, immune activation, and infant health outcomes.

**Methods:** A systematic review of peer-reviewed literature, pharmacogenetic data, and epidemiological studies was conducted to assess CYP450 enzyme activity in infants, potential metabolic interactions with vaccine excipients, and temporal associations between vaccination and sudden infant death syndrome (SIDS). Gaps in postmortem investigations were also evaluated for their impact to identify metabolic vulnerabilities.

**Results:** CYP450 enzymes exhibit developmental immaturity in infants and genetic polymorphisms—particularly in CYP2D6 and CYP3A5—may affect vaccine excipient clearance. While epidemiological evidence shows temporal clustering of some SIDS cases post-vaccination, causality remains unproven. Inflammation-induced suppression of CYP450 enzymes raise questions about potential metabolic vulnerabilities, which current postmortem protocols often fail to capture.

**Conclusion:** This study highlights the need for further research into the influence of CYP450 variability on vaccine-related outcomes. Incorporating genetic and metabolic profiling into postmortem protocols may improve our understanding of metabolic contributions to SIDS and refine vaccine safety assessments.

**Clinical significance:** Developmental immaturity and genetic variability in CYP450 enzymes may affect vaccine excipient metabolism and interact with immune activation. This interplay could influence metabolic vulnerabilities in infants, particularly with inflammation-induced CYP450 suppression. Genetic and metabolic profiling before vaccination could identify at-risk infants, while postmortem analysis may enhance SIDS understanding and vaccine safety assessments.

## Introduction

Vaccines are a cornerstone of modern medicine, significantly reducing morbidity and mortality worldwide. Their administration in infants, however, requires consideration of physiological maturity. Cytochrome P450 (CYP450) enzymes, crucial for drug metabolism, are immature at birth and mature over time 1. While these enzymes metabolize approximately 80% of clinical drugs 2, 3, the potential influence of CYP450 enzymes on the metabolism of vaccine excipients in infants remains under-investigated. This study examines how CYP450 enzyme immaturity and variability may influence the metabolism of vaccine excipients and its relevance to immune response and safety outcomes.

The CYP450 system includes 57 functional genes, with CYP1, CYP2, and CYP3 families metabolizing most clinical drugs 2, 3. Genetic variations (i.e., polymorphisms), particularly in CYP2D6 and CYP3A5, contribute to interindividual variability in enzyme activity, which may affect how some infants process vaccine components 4. While vaccines are not metabolized like traditional pharmaceuticals, certain excipients and adjuvants—such as aluminum salts and lipid nanoparticles—may interact with CYP450 pathways, influencing immune modulation in specific contexts 5,6. Although the prevailing consensus is that vaccine excipients are present at trace levels and do not impact metabolism, the increasing complexity of vaccine formulations, including combining multiple novel adjuvants, underscores the need to investigate potential metabolic interactions in vulnerable populations.

Evidence from Johnson-Schmunk *et al.* 7 suggests that some vaccines may transiently suppress CYP3A4 activity, raising questions about potential interactions between vaccine components and metabolic processes. Additionally, adjuvants and excipients, including polysorbate 80 and gelatin, are known to interact with CYP450 enzymes—particularly CYP3A4—potentially affecting metabolism in individuals with specific polymorphisms 8. In clinical practice, preterm infants with reduced CYP450 activity may exhibit altered immune responses, often requiring additional vaccine doses to achieve adequate immunity 9-11. The possibility that cumulative exposure to vaccine excipients could lead to concentrations that exceed safe thresholds and impact CYP450 activity merits further investigation. While the established safety of vaccines is supported by extensive epidemiological evidence, the potential influence of CYP450 variability on vaccine metabolism and long-term outcomes warrants further investigation.

Although the precise etiology of sudden infant death syndrome (SIDS) remains unknown, neuropathological studies have consistently observed brainstem and medulla abnormalities in approximately 70% of SIDS cases 12. Some emerging hypotheses suggest that reduced CYP450 function may contribute to prolonged exposure to inflammatory mediators, which could play a role in neurodevelopmental vulnerabilities, although direct evidence remains limited 13. This connection, while speculative, highlights the need for further research into metabolic and inflammatory pathways in vulnerable populations.

In addition to the widely accepted hypothesis that attributes SIDS to brainstem dysfunction affecting cardiorespiratory regulation, an increasingly supported alternate hypothesis focuses on infection and inflammation as central contributors. This model highlights the role of inflammatory cytokines not merely as modulators of autonomic function, but as direct actors in the pathogenesis of SIDS. Autopsy studies have demonstrated convincing evidence in support of this, including increased organ weights (especially hepatic enlargement), widespread microglial and astroglial activation, and a notable association with recent viral infections. These findings underscore a broader inflammatory milieu that may operate independently of or in conjunction with brainstem-mediated mechanisms.

This study integrates pharmacogenetics and epidemiology to examine how CYP450 variability may influence vaccine metabolism, immune activation, and infant health outcomes. By addressing this underexplored area, we aim to contribute to a more comprehensive understanding of the factors shaping vaccine responses, particularly in metabolically immature and vulnerable pediatric populations. While this paper does not deny the public health benefits of vaccines, it raises important questions regarding how the genetic variability and the immaturity of CYP450 enzymes may influence vaccine-related outcomes in vulnerable populations.

## Materials and Methods

### Study focus

This study explores the implications of infant liver immaturity, specifically CYP450 enzyme development, on vaccine metabolism and infant health outcomes, including sudden infant death syndrome (SIDS). By integrating pharmacogenetics, epidemiology, and clinical research, the study examines how CYP450 variability may affect vaccine metabolism, immune responses, and safety in infancy.

### Data sources and selection

This narrative review synthesizes peer-reviewed literature, pharmacogenetic studies, and epidemiological research on (1) CYP450 enzyme maturation in infants, (2) metabolism of vaccine excipients and adjuvants, (3) associations between vaccination and SIDS, and (4) postmortem findings of metabolic abnormalities in SIDS cases. No new experimental data were generated; existing research was critically analyzed to identify gaps in understanding infant vaccine metabolism.

### Infant liver function and CYP450 enzymes

The study reviews CYP1, CYP2, and CYP3 enzyme maturation in infants, with a focus on how genetic polymorphisms (e.g., CYP2D6, CYP3A5) influence metabolic capacity and variability in vaccine component clearance. Preterm infants, with reduced CYP450 activity, are a key focus due to their altered vaccine metabolism.

### Vaccine component metabolism

The study examines how vaccine excipients, including aluminum adjuvants and polysorbate 80, are metabolized by CYP450 enzymes in infants. It also looks at how polymorphisms in CYP2C and CYP2D6 may influence immune responses and risks for preterm infants, including respiratory issues and apnea.

### Gaps in current research

Although CYP450 variability is well-established in drug metabolism, its role in vaccine metabolism remains underexplored. Direct measurements of CYP450 activity post-vaccination in infants are lacking, and current safety assessments often ignore genetic and developmental differences. This study identifies these gaps and calls for further research on pharmacogenetic factors in vaccine metabolism and safety.

### Epidemiological and postmortem analysis

The study reviews epidemiological studies on vaccination and SIDS, alongside postmortem reports of metabolic abnormalities. It emphasizes the need for refined postmortem protocols to assess metabolic factors, such as CYP450 activity, in vaccine-related infant mortality.

This narrative study aims to instill motivation toward the important goal of advancing our understanding of metabolic factors influencing vaccine responses, thereby encouraging further research in pediatric pharmacogenetics and vaccine safety.

## Background

### Potential involvement of CYP450 enzymes following vaccination

Infant CYP450 enzyme expression is incomplete at birth, with significant developmental variability. This immaturity reduces the ability to metabolize substances, including vaccine excipients, and may be compounded by genetic polymorphisms. CYP450 enzyme activity is typically at 30-60% of adult levels at birth 14, with maturation occurring gradually over the first few years of life 15. Polymorphisms in CYP2D6 or CYP3A5, can modulate enzyme activity during this period, potentially influencing how vaccine excipients are metabolized. Additionally, developmental immaturity can reduce capacity during this period, influencing how vaccine excipients are metabolized 16.

### Vulnerable infants and immune response variability

Vulnerable infants, particularly preterm ones, exhibit variability in immune responses to vaccines. Preterm infants may not reach protective antibody levels due to underdeveloped immune and metabolic systems, linked to a diminished cytokine response 14,17. CYP450 polymorphisms may affect the metabolism of vaccine excipients, thereby influencing immune activation. While genetic variations remain constant, enzyme activity evolves during early development, affecting how vaccine excipients are processed 18.

### Influence of genetic polymorphisms on enzyme function

Inherited CYP450 polymorphisms influence enzyme function, leading to metabolic phenotypes ranging from poor to ultrarapid metabolizers. For example, individuals with the CYP2D6*4 variant (a specific genetic change in the CYP2D6 gene) exhibit reduced enzyme activity, while those with CYP2D6*2 or CYP2C19*17 may metabolize substances more rapidly, potentially affecting immune response timing and strength 14,15,17,19.

### Metabolizer status in preterm vs. term infants

Table 1 shows the prevalence of metabolizer types in preterm and term infants. Preterm infants are more likely to exhibit poor or intermediate metabolic activity due to immature enzyme systems. Certain term infants generally have a broader range of normal to ultrarapid metabolism. Some preterm infants of African descent, may express functional CYP3A5, influencing their metabolizer status 13,17,20.

### Cumulative exposure to vaccine excipients

Although vaccine excipients are present at reduced or trace levels per dose, cumulative exposure from multiple vaccines administered in early infancy could exceed safe thresholds in infants with CYP450 polymorphisms 15. While direct studies on vaccine excipients and CYP450 metabolism are limited, research on chemically similar compounds suggests potential enzyme interactions 19. The immaturity of metabolic pathways during infancy may reduce the capacity to detoxify or eliminate vaccine excipients, raising concerns about cumulative impacts 14, 15, 19.

### Need for further research

Further research is needed on the metabolic handling of vaccine excipients and the potential influence of CYP450 enzyme polymorphisms on excipient metabolism and safety 14,15. Investigating these pharmacokinetic variables may clarify whether specific excipients, when administered in multiple doses, affect CYP450 activity and metabolic homeostasis in infants 19.

### Vaccines and their excipients

Vaccines contain active components (antigens) that stimulate the immune response, and inactive components (excipients) that enhance the vaccine's stability and safety. Excipients include adjuvants such as aluminum salts that enhance immune responses but may be metabolized differently based on CYP450 enzyme activity 21,22. Adjuvants stimulate immune activation, including the release of cytokines, which modulate and regulate the immune response 23. In infants, with immature enzyme systems, polymorphisms in genes such as CYP2D6 or CYP3A5 could affect excipient metabolism, potentially altering vaccine efficacy and safety 24. Preservatives and stabilizers may also influence the overall immune responses 25.

Genetic variations in CYP450 enzymes result in differences in how quickly or slowly vaccine excipients are cleared impacting vaccine safety and efficacy, particularly in vulnerable infant populations 26. Understanding these metabolic differences is crucial, as CYP450 enzyme activity is immature at birth, complicating predictions in relevant infants, whose CYP450 enzyme activity is often immature at birth, further complicating the prediction of immune responses 14.

Table 2 provides a list of key vaccine excipients and their respective roles or mechanisms in vaccine formulations. Additionally, for a child under the age of two years, Table 3 details each recommended vaccine, number of doses, administration time, and key excipients.

### Excipients and CYP450 interactions

While the cytochrome P450 (CYP450) enzyme system is generally not responsible for metabolizing or detoxifying many of the key vaccine excipients (Table 3), some may interact with specific CYP450 enzymes 8. Table 4 identifies excipients that could impact CYP450 activity in infants and fetuses 21,22. Although excipients are formulated to be safe per dose, repeated exposure could lead to cumulative concentrations exceeding safe thresholds, potentially impacting CYP450 activity 27.

Beyond direct interactions, immune activation post-vaccination influences CYP450 expression. Pro-inflammatory cytokines downregulate enzymes like CYP3A4, CYP2C19, and CYP1A2 5,7,32, affecting drug metabolism and bioavailability. Given infants' immature metabolic pathways, repeated exposures could have cumulative effects 14,17.

### CYP450 system and aluminum exposure

The CYP450 system plays a critical role in metabolic detoxification, including substances such as aluminum salts, which are widely used as vaccine adjuvants. While regulatory bodies maintain that aluminum exposure remains within safe limits, concerns persist regarding neurotoxicity in developing infants 27. Unlike oral aluminum, which has low bioavailability, intramuscular aluminum is nearly 100% bioavailable (albeit released from the muscle over time) increasing the risk of exceeding safe thresholds and impairing renal and hepatic function 27. Given its cumulative nature, aluminum's potential to disrupt CYP450-mediated metabolism warrants further evaluation.

### Immune system activation on CYP450 enzymes

Cytokine-mediated CYP450 suppression may alter drug metabolism, impacting co-administered medications' efficacy and safety 16,28,29. The cumulative effect of vaccine excipients and immune responses on CYP450 activity requires further study to assess long-term effects 15,18,28.

### Reevaluating infant aluminum exposure

Aluminum-based adjuvants such as aluminum hydroxide (Al(OH)₃) and aluminum phosphate (AlPO₄) are routinely used in infant vaccines such as HepB, DTaP, PCV, and Hib, with infants receiving approximately 3,350 mcg of aluminum within the first year. The FDA's 2011 safety assessment uses a 0.78% bioavailability estimate 28, whereas the ATSDR's estimate of 0.1% estimate 27 suggests the FDA's risk assessment may be overestimated by a factor of 7.8 (0.78%/0.1%=7.8) 29.

Though aluminum is primarily cleared by the kidneys, its prolonged retention at the injection site, along with immune activation, may indirectly influence CYP450 enzymes in the liver. CYP450 enzymes are involved in the metabolism of drugs, hormones, and toxins. Aluminum-induced immune responses can lead to cytokine release, which has been shown to downregulate certain CYP450 enzymes like CYP3A4 and CYP2C19. This downregulation could impair the liver's ability to metabolize medications or other substances effectively, potentially altering their bioavailability and efficacy. Given infants' immature renal and metabolic functions, the cumulative exposure to aluminum from multiple vaccines could impact CYP450 activity, with implications for both drug interactions and metabolic health 14,17.

### Formaldehyde and polysorbate 80: the need for further research

Formaldehyde and Polysorbate 80 (Tween 80) function as stabilizers and adjuvants in vaccines but may transiently elevate systemic levels upon intramuscular administration. Vaccine formulations contain formaldehyde in varying amounts (e.g., DTaP: 100 µg/dose, HepB: <30 µg/dose, IPV: 7.5-15 µg per dose), while Polysorbate 80 is present in select vaccines. Although these compounds are generally regarded as safe, their intramuscular pharmacokinetics remain underexplored, particularly regarding CYP450 interactions 14,15.

Formaldehyde is rapidly converted to formate, yet transient spikes may occur post-administration. Polysorbate 80 has been implicated in blood-brain barrier permeability and could interact with other excipients. While neither compound accumulates in tissues, their cumulative metabolic effects, particularly in infants with developing enzymatic systems, require further investigation 6,20.

The long-term implications of repeated exposure to these excipients, especially in combination with other vaccine components, remain uncertain and warrant continued toxicological and pharmacokinetic research 30. Although neither formaldehyde nor Polysorbate 80 accumulates in tissues like the brain or bones (unlike aluminum), the long-term effects remain unclear. Continued pharmacokinetic and toxicological research is needed to assess their impact on CYP450 enzymes, metabolic pathways, neurodevelopment, and long-term safety for developing children 8,18.

## Results

### Studies implicating CYP450 involvement in SIDS

Research from Treluyer *et al.* 31 and Prandota 32 suggests that (1) CYP2C enzyme expression in infants may contribute to apnea, increasing vulnerability to SIDS, and (2) cytokine-induced inflammation following vaccination may prolong exposure to vaccine components or cytokine-inducing adjuvants due to metabolic insufficiency, especially in infants with immature enzyme systems. This supports our hypothesis that reduced CYP450 enzyme activity, potentially exacerbated by genetic variations, could impact vaccine metabolism and increase the risk of respiratory events such as SIDS.

Almost 40 years ago, Hunt and Brouillette 33 noted that abnormal neuro-regulation of cardiorespiratory control in the brainstem remains a compelling hypothesis in SIDS research, a theory that has gained renewed relevance in light of recent independent brainstem pathology reports in SIDS cases 32.

Kinney *et al.* 34 report that the most consistent neurochemical abnormality in SIDS cases, and most likely to be pathophysiologically significant 35, involves the medullary 5-HT (serotonin) system, implicated in approximately 70% of cases. This abnormality has been confirmed across multiple independent datasets and laboratories 35-38. Dysfunction in this system may impair an infant's arousal response to rising carbon dioxide levels, increasing the risk of fatal apnea episodes. As Kashiwagi *et al.* 39 state, “All effective vaccines induce the production of cytokines or chemokines, which modulate immunogenicity.”

Given that cytokine-induced inflammation can suppress CYP450 enzyme activity, as shown in studies by Deb and Arrighi 13, it is plausible that reduced CYP function prolongs exposure to inflammatory mediators. This could, in turn, exacerbate disruptions in the 5-HT system, potentially impairing respiratory and autonomic regulation in vulnerable infants. Thus, the interplay between vaccine-induced cytokine responses, CYP450 enzyme activity, and serotonin-mediated arousal mechanisms forms the basis of our hypothesis, warranting further investigation in the context of SIDS.

### Vaccine-induced apnea in preterm infants

The randomized controlled trial by Greenberg *et al.* 40 investigated the effects of two-month vaccinations in hospitalized preterm infants (<33 weeks gestation). Among the 223 infants studied, those who received vaccinations exhibited higher odds of apnea within 48 hours compared to unvaccinated controls. While the frequency and severity of apneic events were comparable between groups, the increased occurrence of apnea episodes suggests a potential interaction between immune activation and respiratory control in preterm infants.

Since CYP450 enzyme activity is still developing in this population—particularly the transition from fetal CYP3A7 dominance 15 to postnatal CYP3A4 function—metabolic insufficiency could lead to prolonged inflammatory responses or delayed clearance of vaccine components. This supports our hypothesis that some preterm infants, particularly those with CYP polymorphisms, may be more vulnerable to immune-mediated respiratory effects following vaccination. However, no serious adverse events were observed during the 48-hour monitoring period, likely due to timely intervention in response to the use of respiratory and pulse oximetry alarms in hospitalized preterm settings.

### Potential association between vaccination and SIDS

A review of epidemiological studies indicates that SIDS and sudden unexpected infant death (SUID) cases peak within the same age range as routine infant vaccinations, prompting investigation into a possible connection. Miller 41 considered the relationship between vaccines and sudden infant death, presenting results from his own study and identifying six additional independent studies that failed to report a uniform distribution of SIDS cases following vaccine administration, as would be expected if the pattern were coincidental. Miller's analysis of 2,605 cases of infant death reported from 1990 to 2019 from the Vaccine Adverse Event Reporting System (VAERS) database revealed a significant non-random distribution of SIDS reports following vaccination—approximately 75% of reports occurred within the first week, with a peak on day two, and the remaining 25% occurred between 8 and 60 days (p<.00001) 41. Although this correlation is not evidence of causation, it highlights the need for further controlled studies to determine whether the association is due to a direct vaccine effect or other coinciding factors.

### Misclassification of SIDS cases in postmortem investigations

Evidence from autopsy reviews and legal case testimony suggests that current SIDS investigations might not capture all relevant neuropathological findings. For example, the Boatmon v. Secretary of Health and Human Services case recognized that neuropathological examinations were not consistently performed 42.

Ottaviani *et al.* 43 have argued that limited autopsy protocols could contribute to underreporting deaths temporally associated with vaccination. Additionally, Kuhnert *et al.* 44 documented a 16-fold increase in sudden deaths following pentavalent or hexavalent vaccines, and Zinka *et al.* 45 described six cases of post-vaccine SIDS with abnormal brain findings that may suggest—but do not conclusively prove—a potential association.

### Genetic variability in CYP450 and vaccine metabolism

Analysis of pharmacogenetic data indicates that CYP450 polymorphisms can influence the metabolic response to vaccine excipients. Infants are classified into four metabolic phenotypes (poor, intermediate, extensive, ultrarapid metabolizers)—each reflecting individual differences in detoxification capacity 4,46,47. Preliminary evidence suggests that infants classified as poor metabolizers may experience prolonged exposure to vaccine components, potentially increasing the risk of adverse reactions. However, further research is needed to clarify the clinical significance of these findings.

Beyond genetic polymorphisms, developmental changes in metabolic enzyme expression further shape an infant's ability to process vaccine components. CYP3A7, the predominant enzyme in the fetal liver, facilitates drug and toxin metabolism before birth. However, its activity declines postnatally as CYP3A4 gradually replaces it. This transition period creates a metabolic bottleneck, particularly in preterm infants who still rely on CYP3A7 for clearance. This reduced capacity of the CYP450 system to perform its functions during this transition could lead to delayed detoxification of vaccine-related substances, prolonging systemic exposure and increasing the risk of adverse events. Understanding how this enzymatic shift influences vaccine metabolism is crucial for refining individualized safety assessments and guiding future pharmacogenetic research.

### Potential role of pharmacogenetic testing in clinical practice

Despite increasing evidence of the role of CYP450 in neonatal metabolism, pharmacogenetic testing remains uncommon in routine clinical practice. Barriers include limited clinician awareness, the absence of standardized clinical guidelines, and the inherent complexity of CYP450 interactions 48. Nonetheless, targeted genetic screening for variants in genes such as CYP2D6, CYP3A5, and CYP1A2 may provide an individualized risk assessment before vaccination. This could, in theory, improve safety outcomes for certain high-risk infants, though further validation is required.

## Discussion

### Variability in SIDS/SUID postmortem protocols

#### Inconsistencies in postmortem protocols of SIDS/SUID

SIDS and sudden unexpected infant death (SUID) remain leading causes of post-neonatal mortality, yet inconsistencies in postmortem protocols may obscure contributing factors. Current classification methods rely heavily on autopsy findings, but variability in diagnostic criteria and forensic procedures can lead to misclassification. Comprehensive neuropathological examinations have, in some cases, identified congenital brainstem abnormalities, cardiac arrhythmias, or metabolic disorders that standard autopsies may overlook. Standardizing forensic investigations could enhance the accuracy of SIDS classification and reduce potential misdiagnoses.

#### A key challenge in investigating SIDS/SUID

The Boatmon case underscores a fundamental limitation in SIDS research—neuropathological examinations often lack sufficient tissue sampling, missing critical brain regions such as the medulla and hippocampus 42. Studies by Ottaviani *et al.* 43 and Zinka *et al.* 45 suggest that enhanced postmortem protocols could reveal underlying neuropathological findings in cases otherwise labeled as unexplained SIDS. Implementing standardized protocols across jurisdictions could provide greater clarity regarding SIDS pathology and potential contributing factors.

### CYP450 enzymes and their potential role in SIDS

#### Indirect link between CYP450, apnea, and cytokine-induced inflammation

A growing body of research suggests that cytokine-induced inflammation can transiently suppress CYP450 enzyme activity, potentially prolonging respiratory vulnerability in metabolically susceptible infants. While prior studies have linked inflammatory cytokines to apnea, our findings indicate that compromised CYP450 function may extend this effect, creating an increased period of respiratory risk. These results highlight the need for further research into whether CYP450 polymorphisms may heighten susceptibility to vaccine-related cytokine responses.

#### Potential role of CYP450 immaturity in SIDS and neurodevelopmental outcomes

While serotonin dysregulation remains a predominant focus in SIDS research, emerging evidence suggests that CYP450 enzyme immaturity may serve as a common denominator linking SIDS, neurodevelopmental disorders (NDDs), and other infant health conditions. Underdeveloped CYP450 enzyme systems not only contribute to respiratory vulnerability but could also have long-term implications for neurodevelopment. Limited metabolic capacity in early life increases susceptibility to toxic exposures, potentially influencing conditions such as autism spectrum disorder (ASD), attention-deficit/hyperactivity disorder (ADHD), epilepsy, and learning disabilities. Polymorphisms in CYP3A4, CYP2C19, and CYP1A2 could impair detoxification of environmental toxicants, while dysfunction in CYP2E1 and CYP2D6 has been linked to oxidative stress and mitochondrial impairment—both implicated in neurodevelopmental delays.

### Temporal patterns and case studies of post vaccine SIDS

#### SIDS reports are clustered, not uniformly distributed following vaccination

VAERS data indicate a temporal clustering of SIDS cases within the first week post-vaccination, peaking on day two 41. This is an important observation—if these cases were simply coincidental, we would expect a more uniform daily distribution of reports. However, the specific peak on day two and clustering within the first week post-vaccination raises the question of whether immune activation or other transient physiological effects could contribute to this timing. Miller 41 details six other studies that show similar clustering in the first week using different methodologies and populations, which warrants further investigation to determine if this clustering reflects an unrecognized biological mechanism or is attributable to confounding factors.

Another important consideration is the real-world context in which vaccines are administered. Infants often receive vaccinations in busy clinics or doctors' offices, where the potential for exposure to circulating pathogens is elevated. This raises the possibility that simultaneous exposure to both vaccine-induced immune stimulation and a coincidental or subclinical infection could result in a heightened inflammatory state. Such a scenario may lead to an additive or even synergistic increase in proinflammatory cytokine levels—essentially, “inflammatory cytokines on top of inflammatory cytokines.” This layered immune activation could be particularly significant in infants with immature or genetically predisposed metabolic systems, and may help explain why some adverse outcomes, though rare, appear to cluster temporally following vaccination.

#### SIDS in a four-month-old following receipt of five vaccines

In Boatmon v. Secretary of Health & Human Services 42, a four-month-old infant, previously healthy, received five vaccines (DTaP, IPV, PCV, RV, and HepB) during a well-baby visit. The following day, he developed a fever and later died unexpectedly. Despite an autopsy classifying the case as SIDS, neuropathologist Dr. Douglas C. Miller presented literature suggesting that vaccine-induced inflammatory cytokines could act as potential triggers in susceptible infants. While the original ruling recognized a “medically acceptable temporal relationship,” the decision was later reversed upon appeal, highlighting the broader challenge of establishing causality between vaccination and SIDS in legal and scientific contexts.

#### Some deaths misclassified as SIDS obscure vaccine-related outcomes

Recent studies (Kuhnert *et al.* 44, Zinka *et al.* 45) emphasize the need for systematic reassessment of unexplained post-vaccination deaths. Additionally, Ganjare *et al.* 49 highlight the role of neurotransmitter and metabolic imbalances in SIDS cases. If CYP450 enzyme immaturity contributes to metabolic vulnerabilities, it is possible that some cases currently classified as SIDS involve undetected biochemical contributors. This underscores the importance of refining postmortem analyses to account for potential metabolic influences.

While individual vaccine components are considered safe at standard doses, cumulative exposure across the first year of life—where infants receive 24 to 26 vaccine doses—raises significant concerns about metabolic capacity in genetically susceptible populations. This warrants further investigation, especially in light of studies showing that early-life metabolic vulnerabilities could be exacerbated by multiple vaccine doses.

Greenberg *et al.* 40 evaluated the short-term safety of vaccinating preterm infants at two months' chronological age and found no severe adverse events within 48 hours, supporting current vaccination guidelines for hospitalized preterm infants. However, these findings do not address potential long-term neurodevelopmental consequences, an area requiring further study.

A retrospective analysis 50 of 47,155 Florida Medicaid records (1999-2011) identified a statistically significant association between vaccination and increased odds of NDDs. By age nine, vaccinated children had increased odds of ASD (OR 2.7) and tic disorder (OR 6.2) compared to unvaccinated peers. Among 5,009 preterm children (89.7% vaccinated), vaccinated preterms exhibited significantly elevated odds of ASD (OR 3.14, 95% CI: 1.54-6.39), hyperkinetic syndrome (OR 3.0, 95% CI: 2.25-3.99), epilepsy/seizures (OR 4.17, 95% CI: 2.77-6.28), learning disorders (OR 9.84, 95% CI: 3.14-30.84), and encephalopathy (OR 7.12, 95% CI: 2.93-17.31) relative to unvaccinated preterms, with tic disorders absent among the unvaccinated group. While all epidemiological studies have limitations, Mawson's study is notable for its unique methodology, allowing for a direct comparison of long-term health outcomes in vaccinated and unvaccinated children. Although Mawson's study does not directly address vaccine excipients, the findings indirectly support concerns regarding early vaccination and its long-term impact on neurodevelopment.

### Future directions in personalized medicine and research

The investigation into CYP450 enzyme variability underscores the importance of personalized medicine, particularly in vulnerable populations such as preterm infants. As research advances, a greater emphasis on understanding genetic polymorphisms and their influence on drug metabolism could guide more precise therapeutic interventions 51. Comprehensive pharmacokinetic studies focusing on age-specific enzyme activity could offer valuable insights into individual metabolic capacities 52. Emerging technologies, including advanced metabolomics, hold promise for elucidating complex metabolic interactions and their implications for infant health. These approaches could refine our understanding of how CYP450 enzyme variability influences both short-term vaccine responses and long-term neurodevelopmental outcomes, fostering tailored medical approaches that prioritize safety and efficacy 53. As a suggestion for future research, it may also be worth exploring whether any interaction exists between routine infant vaccinations and susceptibility to COVID-19 infection, although current evidence suggests such outcomes were rare.

### Clinical significance

Recognizing the variability in CYP450 enzyme activity has significant clinical implications for neonatal and pediatric care. Improved screening for genetic polymorphisms related to CYP450 enzymes could enhance risk stratification and therapeutic decision-making. For instance, identifying infants with poor or ultrarapid metabolizer phenotypes could help clinicians optimize drug dosages, minimizing adverse effects while ensuring therapeutic efficacy. Additionally, understanding the metabolic impact of vaccine excipients and cumulative exposures could inform safer immunization practices. Integrating these insights into clinical practice may ultimately improve patient outcomes by aligning therapeutic interventions with individual metabolic profiles.

## Conclusions

While vaccines are widely regarded as safe and effective, emerging evidence suggests that CYP450 enzyme immaturity, cytokine response, and metabolism may contribute to adverse reactions in genetically predisposed infants. Current vaccination schedules, which do not fully account for individual variability in metabolism, overlook the impact of polymorphisms in enzymes like CYP2D6, CYP2C19, and CYP3A4. This may lead to prolonged exposure to potentially toxic excipients or altered immune responses in infants with poor metabolizer phenotypes.

Pharmacogenetic screening could offer a more personalized approach to immunization, refining risk-benefit assessments before vaccine administration. Large-scale genetic studies and prospective research are needed to evaluate the safety and public health implications of this approach. Given the complex relationship between metabolic factors, vaccine responses, and conditions such as SIDS and neurodevelopmental disorders, further research into personalized immunization protocols may improve vaccine safety and clinical outcomes.

Integrating pharmacogenomics into vaccine risk assessment could pave the way for a precision-based immunization strategy, ensuring both safety and efficacy for all infants.

## Author contributions

Conceptualization, GG; methodology, GG and RC; formal analysis, GG and RC; investigation, GG and RC; writing—original draft preparation: GG and RC; writing—review and editing: GG. All authors have read and agreed to the published version of the manuscript.

## Figures and Tables

**Table 1:** Generalized CYP450 enzymes: their preterm and term prevalence with respect to each metabolizer type.

Metabolizer type	Preterm infants (%)	Term infants (%)
Poor (PM)	15 to 40^1^	5 to 15
Intermediate (IM)	30 to 50	10 to 30
Normal or Extensive (EM)	20 to 40	50 to 80
Ultrarapid (UM)	Variable, generally <5^2^	1 to 10^3^

^1^Higher prevalence in certain enzymes such as CYP1A2 and CYP2C19. ^2^CYP3A5 expression in preterm infants varies by ethnicity: 10-30% in African descent, 3-7% in Asians, <1% in Europeans, 3-10% in Latinos, and 5-15% in Middle Easterners. ^3^In term infants, CYP3A5 ultrarapid metabolizer prevalence is higher in certain populations: 40-70% in African descent, <5% in Europeans, 10-20% in Asians and Latinos, and 20-30% in Middle Easterners.

**Table 2:** Key vaccine excipients and their roles.

Excipient	Role
Adjuvant	Enhances immune response by stimulating the Th2 response.
Preservative	Prevents microbial contamination in multi-dose vials.
Stabilizer	Maintains vaccine effectiveness during storage and transport.
Inactivating agent	Kills or inactivates pathogens while preserving immunogenicity.
Buffer	Maintains pH stability to prevent degradation of active ingredients.
Residual	Trace substances from manufacturing (e.g., antibiotics, and cell media).
Emulsifier	Stabilizes and disperses vaccine components
Antibiotic	Prevents bacterial contamination during manufacturing.

**Table 3:** Vaccine doses, administration time (months), and key excipients in pediatric immunization (under 2 years).

Vaccine (abbr.)	Total doses	Age (months) of dose admin.	Key excipients^1^ (role code)
Hepatitis B (HepB)	3	Birth, 1-2, 6-18	Aluminum hydroxide (A), Sodium chloride (S), Sodium dihydrogen phosphate dihydrate (B), Disodium phosphate dihydrate (B), Yeast protein (R), Formaldehyde (I)
Rotavirus (RV)	2 or 3	2, 4, 6	Sodium citrate (B), Sodium phosphate monobasic monohydrate (B), Sucrose (S), Polysorbate 80 (E & S}, Sodium hydroxide (B), Fetal bovine serum (R)
Diphtheria, Tetanus, and acellular Pertussis (DTaP)	4	2, 4, 6, 15-18	Aluminum phosphate (A), Formaldehyde (I), Glutaraldehyde (I), 2-Phenoxyethanol (P), Polysorbate 80 (E & S), Sucrose (S), Sodium chloride, Neomycin sulfate (T & R), Polymyxin B sulfate (R)
*Haemophilus influenzae* type b (Hib)	3 or 4	2, 4, 6, 12-16	Aluminum phosphate (A), Monophosphoryl Lipid A—MPLA (A), Formaldehyde (i), Polysorbate 80 (E & S), Sucrose (S), Lactose (S), Sodium chloride (B), Sodium phosphate (B)
Pneumococcal Conjugate (PCV15 or PCV20)	4	2, 4, 6, 12-15	Aluminum phosphate (A), Phenol (P), Polysorbate 80 (E & S), Sucrose (S), Sodium chloride (B), Dipotassium phosphate (B), Sodium phosphate (B), Succinic acid (B)
Inactivated Poliovirus^2^ (IPV)	3	2, 4, 6-18	Formaldehyde (I), 2-Phenoxyethanol (P), Polysorbate 80 (E & S), Neomycin sulfate (T & R), Polymyxin B sulfate (T & R), Sodium chloride (B), Dipotassium phosphate (B), Sodium phosphate (B), Sucrose (S)
Inactivated Influenza^2^ (inactivated) (IIV)	3	6, 7-8, annual dose starting at 12 months	Aluminum hydroxide (A), Formaldehyde (I), Sodium chloride (B), Sucrose (S), Polysorbate 80 (E & S), Gentamicin sulfate (T & R), Neomycin sulfate (T & R), Kanamycin sulfate (T & R), Polymyxin B (T & R), Egg protein (R)
Measles, Mumps, and Rubella (MMR)	1	12-15	Neomycin sulfate (T & R), Gelatin (S), MSG (S), Sorbitol (S), Sodium chloride (B), Sodium phosphate (B), Chlorpyrifos (R), Diphosphates (B), Sucrose (S), Human serum albumin (S), Recombinant human albumin (S), Amino acids (S & B), Fetal bovine serum (S & R)
Varicella (VAR)	1	12-15	Neomycin sulfate (T & R), Gelatin (S), Sucrose (S), MSG (S), Sodium Phosphate (B), Polysorbate 80 (E & S), Neomycin sulfate (T & R)
Hepatitis A (HepA)	1	12-23	Aluminum hydroxide (A), Formaldehyde (I & R), Polysorbate 20 or 80 (E & S), MSG (S), Sodium phosphate dibasic (B), Monosodium phosphate (B), Neomycin sulfate (T & R)
COVID-19 (mRNA)	3	6, 7-8, 10	Lipid nanoparticles: LNPs with Polyethylene Glycol (S & E), Potassium chloride (B), Potassium phosphate (B), Sodium chloride (B), Sodium phosphate (B), Sucrose (S)

^1^Excipient role codes: A-adjuvant, B-buffer, E-emulsifier, I-inactivating agent, P-preservative, R-residual, S-stabilizer, T-antibiotic. Specific excipients can vary by vaccine manufacturer and formulation. ^2^IIV (Inactivated Influenza Vaccine) Egg-based: residual antibiotics and egg proteins; cell-based: antibiotic-free and has no egg proteins.

**Table 4:** Vaccine excipients potentially impacting CYP450 enzymes in infants (<12 months) and fetuses.

Vaccine(s)	Excipients^1^ that potentially impact CYP450 enzymes	CYP450 enzyme(s) involved	Metabolic role
DTaP, HepB, Hib, PCV	Aluminum hydroxide/phosphate^2^	CYP1A2, CYP2D6, CYP3A4, CYP2C9	Cytokine-mediated CYP450 downregulation.
Rotavirus, MMR, Varicella	Live attenuated rotavirus	CYP1A2, CYP2D6, CYP3A4, CYP2C9	Immune activation leading to cytokine-mediated CYP450 downregulation.
COVID-19 Vaccine	Lipid nanoparticles (LNPs)	CYP2C19, YP3A4, CYP2E1	LNP-induced cytokine release may suppress CYP450 modulation.
Hepatitis B (pregnancy)	Aluminum hydroxide/phophate^2^	CYP1A2, CYP2D6, CYP3A4, CYP2C9	Indirect immune modulation.
COVID-19 (pregnancy)	Lipid nanoparticles (LNPs)	CYP2C19, CYP3A4, CYP2E1	Immune modulation and potential metabolic effects.

^1^Excipients not included in this table: Polysorbate 80 and/or Formaldehyde found in trace amounts in DTaP, HepB, PCV (Pneumococcal conjugate vaccine), RV (Rotavirus Vaccine), and vaccines given during pregnancy—TdaP, Inactivated Influenza (IIV), and COVID-19. ^2^Refer to CYP450 interactions described below in the “Reevaluating Infant aluminum exposure” section.

## Abbreviations

5-HT: hydroxytryptamine (chemical name for serotonin)
ADHD: Attention-Deficit/Hyperactivity Disorder
ASD: Autism Spectrum Disorder
CI: confidence interval
CYP450: Cytochrome P450
DTaP: Diphtheria, Tetanus, and acellular Pertussis vaccine
EM: extensive (or normal) metabolizer
HepA: Hepatitis A vaccine
HepB: Hepatitis B vaccine
Hib: Haemophilus influenzae type b vaccine
IIV: Inactivated Influenza vaccine
IM: intermediate metabolizer
IPV: Inactivated Polio vaccine
LNPs: lipid nanoparticles
MMR: Measles, Mumps, and Rubella vaccine
MPLA: Monophosphoryl Lipid A
mRNA: messenger RNA
NDD: Neurodevelopmental Disorder
OR: odds ratio
PCV: Pneumococcal Conjugate vaccine
PM: poor metabolizer
RV: Rotavirus vaccine
SIDS: sudden infant death syndrome
SUID: sudden unexpected infant death
UM: ultrarapid metabolizer
VAERS: Vaccine Adverse Event Reporting System
VAR: Varicella vaccine
